# Identification of fertility-related genes for maize CMS-S via Bulked Segregant RNA-Seq

**DOI:** 10.7717/peerj.10015

**Published:** 2020-09-30

**Authors:** Xiner Qin, Wenliang Zhang, Xue Dong, Shike Tian, Panpan Zhang, Yanxin Zhao, Yi Wang, Jianbing Yan, Bing Yue

**Affiliations:** 1 National Key Laboratory of Crop Genetic Improvement, Huazhong Agricultural University, Wuhan, China; 2 Beijing Key Laboratory of Maize DNA Fingerprinting and Molecular Breeding, Maize Research Center, Beijing Academy of Agriculture and Forestry Sciences, Beijing, China; 3 Industrial Crops Research Institution, Heilongjiang Academy of Land Reclamation of Sciences, Haerbin, China

**Keywords:** BSR-Seq, Differentially expressed genes, Fertility restoration, CMS-S, Maize

## Abstract

Cytoplasmic male sterility (CMS) is extensively used in maize hybrid production, and identification of genes related to fertility restoration for CMS is important for hybrid breeding. The fertility restoration of S type CMS is governed by several loci with major and minor effects, while the mechanism of fertility restoration for CMS-S is still unknown. In this study, BSR-Seq was conducted with two backcrossing populations with the fertility restoration genes, *Rf3* and *Rf10*, respectively. Genetic mapping via BSR-Seq verified the positions of the two loci. A total of 353 and 176 differentially expressed genes (DEGs) between the male fertility and male sterile pools were identified in the populations with *Rf3* and *Rf10*, respectively. In total, 265 DEGs were co-expressed in the two populations, which were up-regulated in the fertile plants, and they might be related to male fertility involving in anther or pollen development. Moreover, 35 and seven DEGs were specifically up-regulated in the fertile plants of the population with *Rf3* and *Rf10*, respectively. Function analysis of these DEGs revealed that jasmonic acid (JA) signal pathway might be involved in the *Rf3* mediated fertility restoration for CMS-S, while the small ubiquitin-related modifier system could play a role in the fertility restoration of *Rf10*.

## Introduction

Cytoplasmic male sterility (CMS) is widely used in crop hybrid production and is controlled by both mitochondrial sterile and nuclear restorer genes (Horn, Gupta & Colombo, 2014; Bohra et al., 2016). Besides the great commercial importance for crop hybrid breeding, fertility restoration genes and CMS provide an opportunity to understand the interactions between proteins encoded by mitochondrial CMS-associated genes and nuclear fertility restorers. Thus, it is of great interest to understand the mechanisms of fertility restoration in CMS plants. Fertility restoration of CMS was mainly controlled by pentatricopeptide repeat (PPR) proteins, and genes encoding an aldehyde dehydrogenase, a glycine-rich protein, a pepetidase of the M48 family, and a WD40-like repeat protein were also found to be involved in the fertility restoration of CMS in crops (Chen & Liu, 2014; Qin et al., 2016).

There are three types of CMS in maize, T (Texas), C (Charrua), and S (USDA). Among them, the S-type CMS (CMS-S) is the largest group with a wide range of cytoplasmic sources. In CMS-S of maize, the 1.6 kb transcript of the R region of mitochondrial genome contains the chimeric gene *orf355-orf77*, which is thought to be related to male sterility (Wen & Chase, 1999; Matera et al., 2011). The nuclear fertility restorer, *Rf3*, could restore its fertility through affecting the transcription of the chimeric gene in the male-sterile lines (Zabala, Gabay-Laughnan & Laughnan, 1997). *Rf3* was mapped on the long arm of chromosome 2 (Kamps & Chase, 1997; Laughnan & Gabay, 1978; Zhang, Wang & Zheng, 2006; Xu et al., 2009). Besides *Rf3*, other loci for fertility restoration to CMS-S, such as *RfIII* and *RfIV* (Laughnan & Gabay, 1973), *Rf9* (Gabay-Laughnan et al., 2009) have also been reported. With a genome-wide association study (GWAS), a total of 19 significant loci were identified controlling the fertility restoration to CMS-S, and function analysis of the predicted candidate genes implied that several mechanisms might play roles in fertility restoration to CMS-S (Feng et al., 2015).

RNA-Seq based on next-generation sequencing is extensively used to identify critical genes and pathways associated with agronomically important traits. In recent years, a number of fertility-related genes for CMS have been identified via RNA-Seq by comparing gene expression between CMS and their fertility restoration lines (Liu et al., 2013; Suzuki et al., 2013; An et al., 2014; Han et al., 2017). Bulk Segregant RNA-Seq (BSR), which is a combination of the methods of bulked segregant analysis (BSA) and RNA-Seq, was used to map genes and identify differential expression genes between the bulks quickly (Liu et al., 2012; Hill et al., 2013). In recent years, BSR-Seq has been utilized in genetic mapping and identification of candidate genes related to different agronomic traits in maize (Feng et al., 2015; Li et al., 2013). In 2016, six genes, which encode serine/arginine-rich protein 45, ATP-dependent RNA helicase, leucine-rich repeat extensin-like protein, putative SPRY-domain family protein, and mitochondrial uncoupling protein, were identified potentially associated with minor-effect fertility restoration loci of CMS-S in maize via BSR-Seq (Su et al., 2016).

In this study, BSR-Seq was conducted using a BC1 and a BC2 populations harboring the major restorer of CMS-S, *Rf3,* and a newly identified fertility restoration locus, *Rf10*, respectively. The objectives as followings: mapping the genetic regions of the two fertility restoration loci; identification of differential expression genes (DEGs) that might be involved in male fertility; and comparing and function analysis of the DEGs identified in the two populations. Results in this study would provide useful information to further understand the genetic basis and mechanism of fertility restoration for CMS-S in maize.

## Materials & Methods

### Plant materials

A BC1 population, S-Mo17^*Rf3Rf3*^/Mo17//Mo17 (P1) and a BC2 population, S-Mo17^*Rf3Rf3*^/W2// Mo17///Mo17 (P2) were grown in 2017 in the experimental station of Beijing Academy of Agriculture and Forestry Sciences, Beijing, China. S-Mo17^*Rf3Rf3*^ is a S type CMS line with the nuclear background of maize inbred Mo17, and S-Mo17^*Rf3Rf3*^ is a near isogenic line of S-Mo17^*Rf3Rf3*^ harboring the main restoration gene, *Rf3*. The maize inbred W2 is an inbred with another restoration gene, *Rf10*.

About half of the anthers were collected from the tassels about to exsert from the upmost leaves at room temperature, and stored in a 10 × volume RNA wait solution (Solarbio, Beijing). The remaining tassels were kept for identification of male fertility.

### Phenotyping

Male fertility of each individual was investigated according to the method described as Feng et al. (2015). The level of anther exsertion and dehiscence degree was investigated in field with scales ranged from 1 (no anther exserted and no pollen dehisced) to 5 (anthers exserted and dehisced absolutely). In addition, spikelets close to the newly flowered spikelets on the middle of the tassel were sampled and fixed in a solution with 75% ethanol and 25% acetic acid (v/v). Pollens were squeezed out from the anthers and stained with an iodine-potassium iodine solution (containing 0.5% (w/v) iodine and 1% (w/v) iodine potassium). Then, fertility of the pollens was examined with a light microscope (Olympus IX71, Japan) at 100× magnification.

### RNA extraction, BSR-Sequencing, alignment and SNP discovery

According to the fertility of the plants, 30 fertile (with exserted anthers) and 30 sterile (without exserted anther) samples of each population were selected for BSR-Seq. Anthers were separated from each sample, then RNA of the anthers was extracted using Trizol (Invitrogen, USA). After determining the integrity and concentration by Agilent2010 (Agilent, Santa Clara, CA), the RNA was uniformly mixed into fertile and sterile pools, respectively. Transcriptome of the two pools was sequenced using an Illumina HiSeq2500 (Illumina, San Diego, CA) at Biomarker Technology Co., LTD (Beijing, China).

Quality control, alignment, and SNP discovery of the sequences was carried out by DATA2Bio® (https://www.data2bio.com/) (Liu et al., 2012). The nucleotides of each raw read were scanned for quality checking and trimming. Then the trimmed reads were aligned to the *Zea mays* AGPv3 reference genome (https://maizegdb.org/). Subsequently, SNP calling was conducted using only those reads that uniquely aligned to a single location in the reference genome. The uniquely aligned reads that passed the filtering criteria were used for SNP discovery, and the resulting SNPs were used to conduct QTLs mapping and DEG analysis.

### QTLs mapping and DEG identification

BSR-Seq mapping and DEG analysis was also conducted by DATA2Bio® (https://www.data2bio.com/). A Bayesian approach (Liu et al., 2012) was used to determine the probability of linkage between each high confidence SNP and the restoration gene. Association probability (level) of each SNP >0.05 indicates the SNP is significantly associated with the restoration gene.

Identification of DEGs between the sterile and fertile pools was performed using Fisher’s exact test according to a previous report (Liu et al., 2012). Only informative genes, which required at least 40 total reads summed across the two samples were analyzed. To decrease false discovery rate (FDR), an FDR of 0.01 and a log2 (fold change) of 3 were applied as cutoff to define significant DEGs. Meanwhile, the DEGs identified in one population with FDR less than 0.01 and log2 (fold change) larger than 1 in another population are defined as co-expressed DEGs. GO and KEGG analysis of the DEGs was performed according to the description of two previous reports (Ashburner, Ball & Blake, 2000; Kanehisa, 2004).

### Expression analysis of some DEGs

Eighteen DEGs were selected for validation via qRT-PCR. The anthers were sampled from the tassels about to exsert from the upmost leaves, and the method of RNA extraction was also the same as that for the BSR-Seq analysis. Integrity and concentration of the RNA samples were measured with Agilent2010 (Agilent, Santa Clara, CA). First-strand cDNA was synthesized using *TransScript*® II One-Step gDNA Removal and cDNA Synthesis SuperMix (TransGen Biotech,Beijing,China) based on the manufacturer’s instructions. Primers were designed using the online tool Primer3.0 and the primer sequences are included in Table S2. The qPCR was performed in a 20 µl system for each DEG with the Bio-Rad CFX Manager3.0 system (BIO-RAD, USA). The 20 µl qPCR mixture included 6 µl of diluted cDNA, 10 µl of 2xSybr Green qPCR Mix, and 0.5 µl of each of the 10 *μ*M primers. The amplification was performed as follows: 95 °C for 2 min followed by 40 cycles of 95 °C for 20 s; 62 °C for 20 s and 72 °C for 30 s. The maize Actin gene (*LOC100282267*) was served as the internal control. The Ct value of each sample was calculated, and the relative expression level of each gene was calculated as 2^−ΔΔ^Ct. The experiment was conducted with three biological replicates (each with two technical replicates) for each DEG.

## Results

### Male fertility in the two populations

A total of 195 and 80 individuals in P1 and P2 were grown and investigated, respectively. In P1, anthers in 104 individuals were exserted and dehisced completely, and anthers in 91 individuals were not exserted at all. In P2, anthers in 43 and 37 individuals were exserted and not exserted at all, respectively. Pollen fertility in the un-exserted anthers of the both population was completely sterile. The pollens were dehisced absolutely with fertility about 50% in the exserted anthers of P1, while in the exserted anthers of P2 most of the pollens were dehisced and the pollen fertility was only about 30% (Fig. S1). The ratio of fertile (with exserted anthers) to sterile (without exserted anthers) fits to 1:1 in both of P1 (*χ*^2^ = 0.21, *P* = 0.35) and P2 (*χ*^2^ = 0.46, *P* = 0.50). This indicates that the male fertility was only governed by *Rf3* and *Rf10* in P1 and P2, respectively, and the restoration ability of *Rf3* was stronger than *Rf10*.

### Summary of the BSR-Seq and genome alignment

The sequenced raw reads (http://www.ncbi.nlm.nih/gov/bioproject/PRJNA626059) were scanned for low quality regions and bases with PHRED quality scores of <15 out of 40 were trimmed. Then the trimmed sequence reads were aligned to the reference genome (*Zea mays* AGPv3) using GSNAP (Wu & Nacu, 2010). A summary of the reads that aligned (uniquely and non-uniquely) is provided in Table 1. However, only reads with a single unique alignment were used in subsequent analyses.

**Table 1:** Summary of the BSR sequences.

	Alignments (≥1 Location)	Unique alignments (Single location)
P1-F	2 × 20,160,594 (89.0%)	2 × 18,782,188 (82.9%)
P1-S	2 × 18,003,354 (88.7%)	2 × 17,502,340 (86.3%)
P2-F	2 × 18,719,009 (88.9%)	2 × 18,179,247 (86.3%)
P2-S	2 × 21,230,130 (88.9%)	2 × 20,524,277 (86.0%)

**Notes.**

P1-F, P1-S, P2-F and P2-S are the fertile and sterile pools of the populations of *Rf3* and *Rf10*, respectively.

### SNP discovery and BSR-Seq mapping

SNP discovery was performed using the uniquely aligned reads from all the samples using Data2Bio’s Homozygous and Heterozygous variant discovery models (Ott et al., 2017). For the populations of *Rf3* and *Rf10*, total polymorphic sites identified are 716,225 and 716,923, respectively. Of them, the SNPs with at least 3 reads for the reference allele and 3 for the alternative allele in the fertile pool, and at least 5 reads counts in total for two alleles in the sterile pool were used as input SNPs to identify the locations of the causal loci in the following analyses. Totally 27,297 and 27,893 SNPs were used to mapping *Rf3* and *Rf10*, respectively.

**Figure 1:**
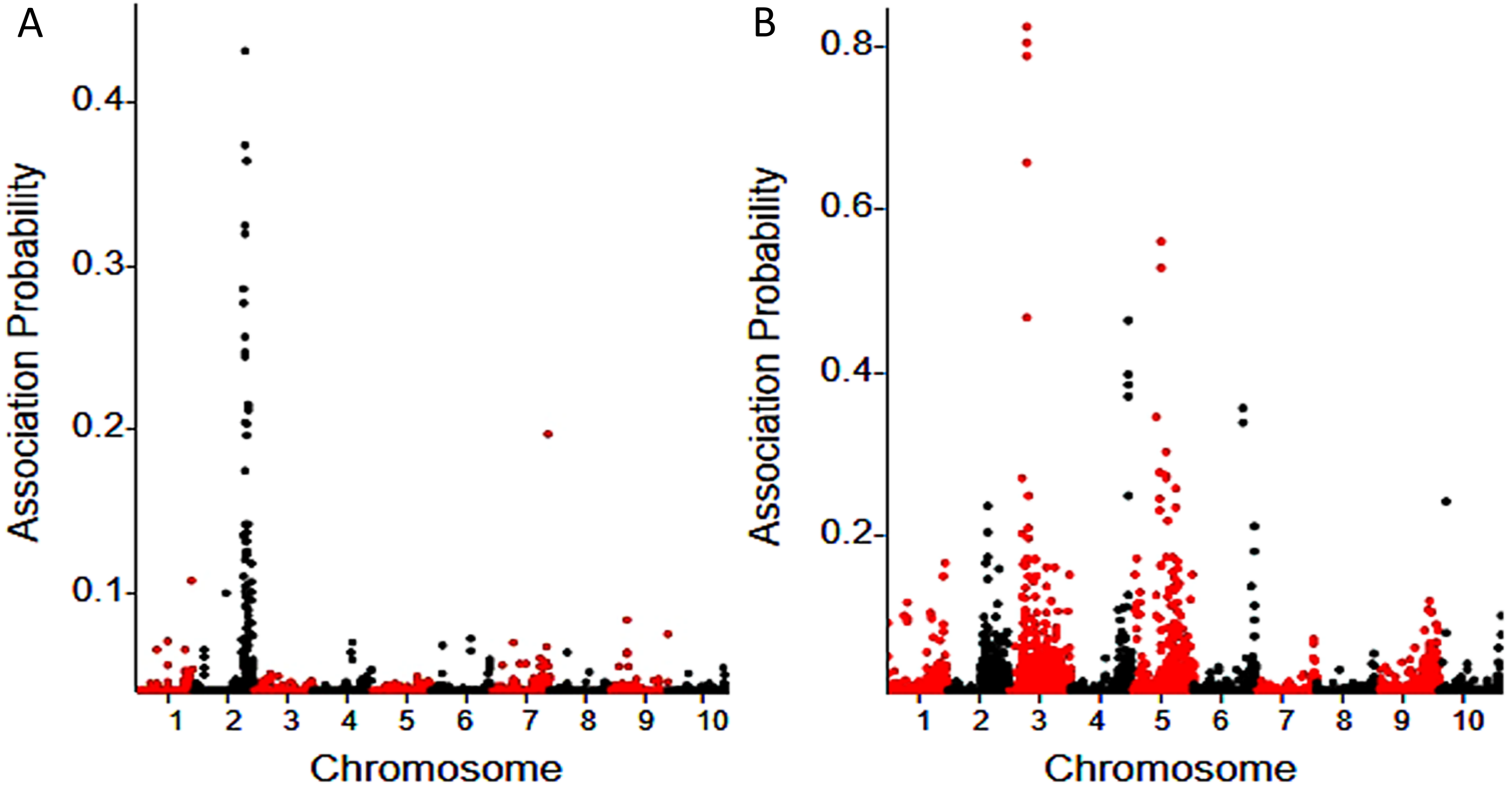
BSR-Seq mapping of *Rf3* (A) and *Rf10* (B).

A Bayesian approach (Liu et al., 2012) was used to determine the probability of linkage between each SNP and the fertility restoration gene. A total of 50 associated SNPs were identified for *Rf3* using a probability cutoff of 0.05, and most (46) of them are located on chromosome 2. Totally 191 associated SNPs were identified for *Rf10*, and 153 of them are located on chromosome 3, 4, 5 and 6 (Fig. 1). *Rf3* was mapped in the interval 227,700,000-229,580,000 (*Zea mays* AGPv3) of chromosome 2, and this is consistent with previous reports (Kamps & Chase, 1997; Laughnan & Gabay, 1978; Zhang, Wang & Zheng, 2006; Xu et al., 2009; Feng et al., 2015). In the population with *Rf10*, the association SNPs with highest probability peaked in the interval of 7,518,726-11,199,114 of chromosome 3 (*Zea mays* AGPv3) (Fig. 1). This indicates that *Rf10* is a new locus different from *Rf3*.

### Differentially expressed genes

**Figure 2:**
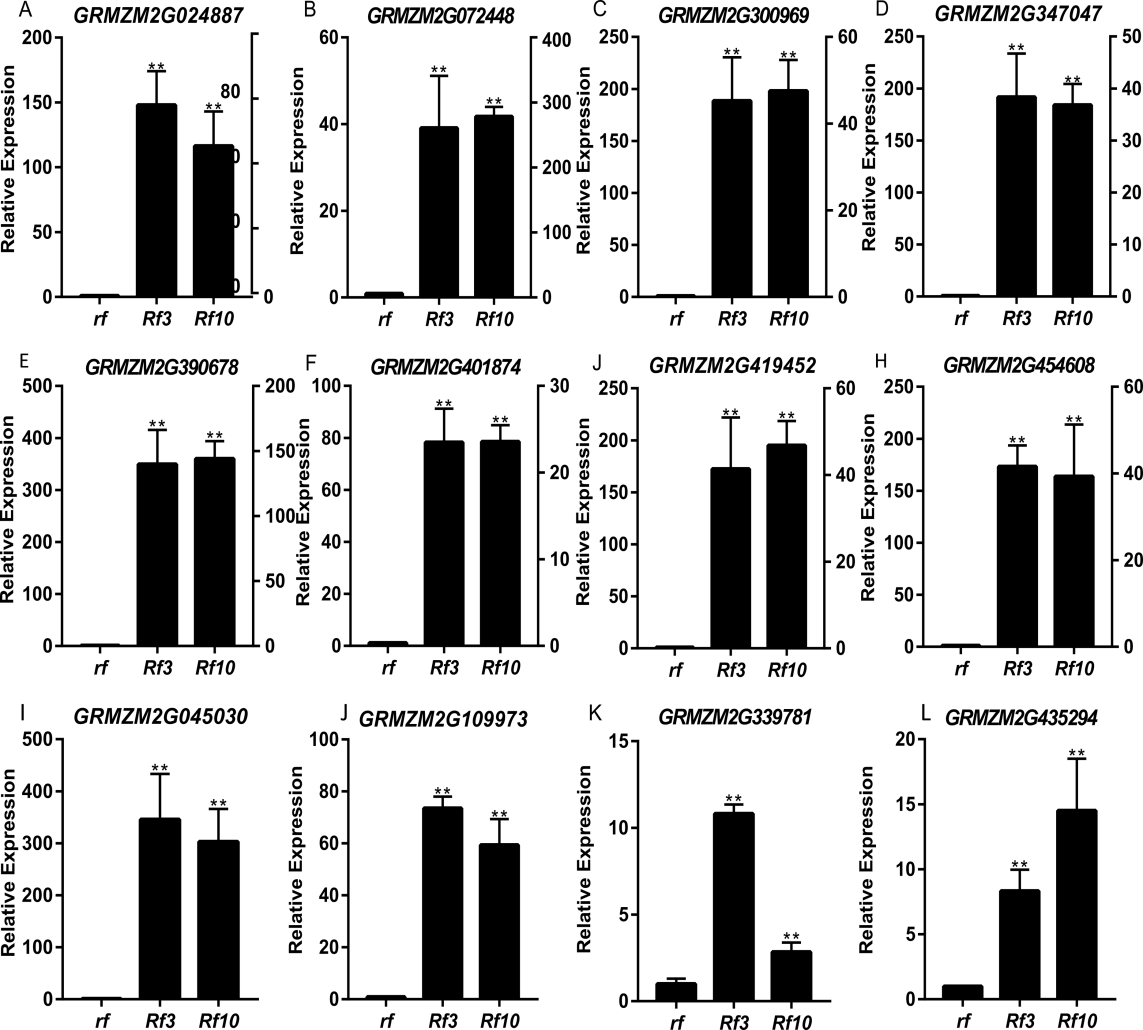
Quantitative RT-PCR confirmation of the 12 DEGs up-regulated in the fertile plants of the two populations. Genotypes of *rf*, *Rf3*, and *Rf10* are S-CMS^*rf3rf3rf10rf10*^, S-CMS^*Rf3rf3rf10rf10*^, and S-CMS^*rf3rf3Rf10rf10*^, respectively. *, ** represents the difference of relative expression between *Rf3* or *Rf10* and *rf* are significant at the level of *P* < 0.05 and *P* < 0.01, respectively (*n* = 3). *rf* and *Rf3* refer to the left *Y*-axis, *Rf10* to the right *Y*-axis (A–H). A–L show that these 12 DEGs are significantly up-expressed in the fertile plants of the two populations.

**Figure 3:**
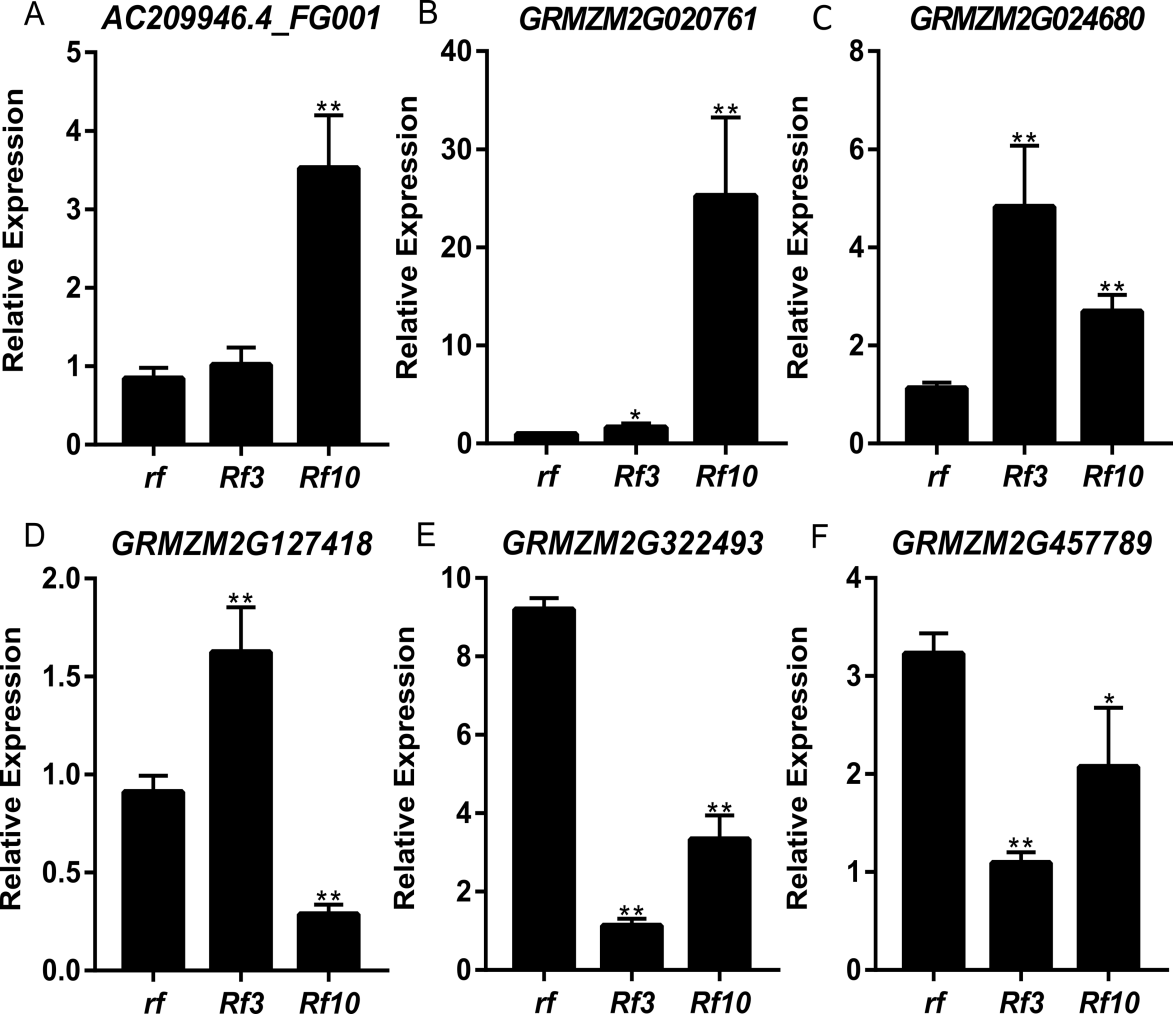
Quantitative RT-PCR confirmation of the 6 DEGs specifically up-regulated or down-regulated in the fertile plants of P1 or P2. Genotypes of *rf*, *Rf3*, and *Rf10* are S-CMS^*rf3rf3rf10rf10*^, S-CMS^*Rf3rf3rf10rf10*^, and S-CMS^*rf3rf3Rf10rf10*^, respectively. *, ** represents the difference of relative expression between *Rf3* or *Rf10* and *rf* are significant at the level of *P* < 0.05 and *P* < 0.01, respectively (*n* = 3). A–F show that these 6 DEGs are specifically up-regulated or down-regulated in the fertile plants of P1 or P2.

DEGs were identified between the fertile and sterile pools of the population with *Rf3* (P1) and *Rf10* (P2), respectively. A total of 353 and 176 genes were differentially expressed between the fertile and sterile pools in P1 and P2, respectively (Table S1). Of them, 284 and 171 DEGs were up-regulated in the fertile pool of P1 and P2, respectively. The DEGs identified in one population with FDR less than 0.01 and log2 (fold change) larger than 1 in another population are considered to be have the same expression pattern in the both populations. Majority (265) of the DEGs were up-regulated in the fertile plants of the two populations simultaneously, and 227 of them were specifically or mainly expressed in pollens or anthers according to MaizeGDB qTeller (http://qteller.maizegdb.org). Of the 104 DEGs specifically identified in P1, 69 and 35 DEGs were down-regulated and up-regulated in the fertile plants, respectively. For the 12 DEGs only differentially expressed in P2, seven DEGs were up-regulated in the fertile pool. Thirty-seven of the specific DEGs identified in P1 and three of the specific DEGs identified in P2 were specifically or mainly expressed in pollens or anthers according to MaizeGDB qTeller. Expression of twelve DEGs co-expressed in the two populations was verified via qPCR. The twelve DEGs were significantly and strongly expressed in the fertile plants in both populations, and this agrees well with the BSR-Seq data (Fig. 2, Table S1, and Fig. S2). In addition, expression of the two DEGs specifically up-regulated in the fertile plants of P2 (*AC209946.4_FG001* and *GRMZM2G020761*), and a DEG only identified in P1 (*GRMZM2G024680*) was verified via qPCR (Fig. 3, Table S1, and Fig. S2). Although the expression of *AC209946.4_FG001* and *GRMZM2G020761* in the fertile plants of P1 and the expression of *GRMZM2G024680* in the fertile plants of P2 was also significant higher than that in the sterile ones, the expression level increased less than or about two times. Expression of a DEG specifically down-regulated in the fertile plants of P2 (*GRMZM2G127418*) and two DEGs identified down-regulated only in P1 (*GRMZM2G457789* and *GRMZM2G322493*) was also verified via qPCR (Fig. 3, Table S1, and Fig. S2). Comparing with the CMS lines, the expression of *GRMZM2G457789* and *GRMZM2G322493* in the fertile plants of P2 also decreased, but it was much higher than that in P1 (Fig. 3, Table S1, and Fig. S2). In general, these results indicate the DEGs identified in this study are reliable.

**Figure 4:**
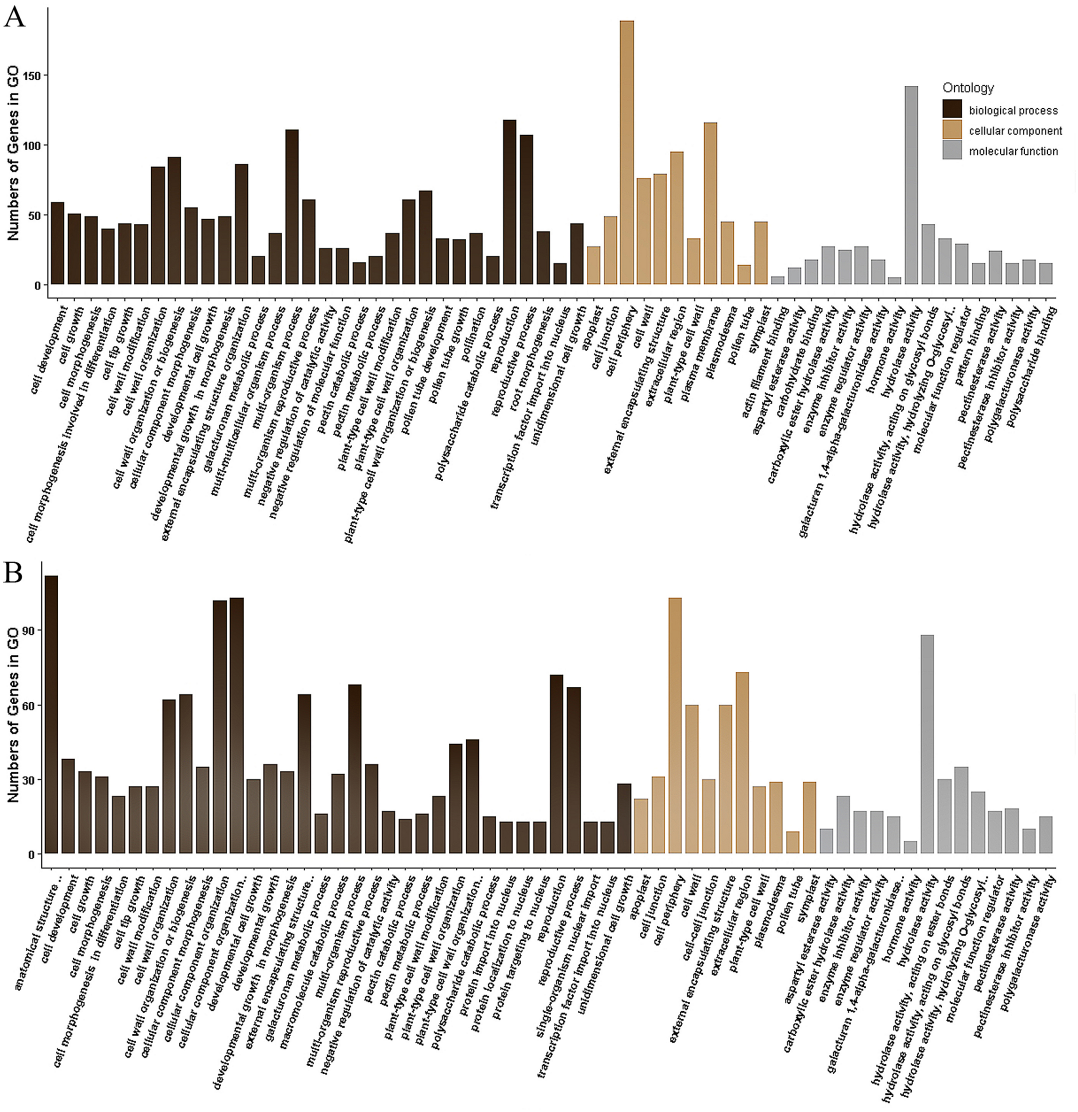
Gene Ontology (GO) classification of the DEGs identified in P1 (A) and P2 (B).

The 355 DEGs identified in P1 were classified into 114 GO groups, including 82, 14 and 18 terms in the main classification of biological process (BP), cellular component (CC), and molecular function (MF), respectively. These DEGs mainly enriched in the groups of multi-organism reproductive process (*N* = 118), and cell wall organization or biogenesis (*N* = 91) of BP; in cell periphery (*N* = 189) and plasma membrane (*N* = 116) of CC; and in hydrolase activity (*N* = 142, including that acting on glycosyl bonds, hydrolyzing O-glycosyl compounds and hydrolyzing carboxylic ester) of MF (Fig. 4A). KEGG analysis of these DEGs revealed that they were mainly involved in three KEGG pathways, including 35 DEGs participated in Metabolic pathways (K01100), 20 DEGs involved in Pentose and glucuronate interconversions (K00040), and eight genes participated in Biosynthesis of secondary metabolites (K01110).

The 173 DEGs identified in P2 could be classified into 121, 11 and 20 terms in BP, CC and MF, respectively. In the main classification of BP, the DEGs mainly enriched in the groups of anatomical structure development (*N* = 112) and cellular component organization or biogenesis (*N* = 103), multi-organism process (*N* = 68), reproductive process (*N* = 67), external encapsulating structure organization (64) and cell wall organization or biogenesis (*N* = 64). In the main classification of CC and MF, GO enrichment of these DEGs is similar to that identified in P1, which enrich in cell periphery (*N* = 103) and hydrolase activity (*N* = 88), respectively (Fig. 4B). A total of 36 DEGs in P2 were annotated to 19 KEGG pathways, and they were mainly associated with the three pathways identified in the P1. There were 30, 19 and 6 DEGs involved in Metabolic pathways (K01100), Pentose and glucuronate interconversions (K00040), and Biosynthesis of secondary metabolites (K01110), respectively.

### Function of the DEGs specifically identified in P1 or P2

Function of the 13 specific DEGs up-regulated in the fertile plants of P1 is unknown. Of the rest 22 specific DEGs, five encode protein kinases. In addition, DEGs encoding a JAZ1 (Jasmonat-zim-domain protein), a 2-oxoglutarate/Fe (II)-dependent dioxygenase, a DEG for ethylene responsive element binding protein (EBP), a thionin, a DnaJ, two P450 proteins, and an oleosin were specifically identified in P1. DEGs encoding mitochondrial ribosomal protein s12, inositol/sucrose transporter, PME/PMEIs, arabinogalactan, UDP-glucosyltransporter and amino acide permease were also specifically up-regulated in the fertile plants of P1.

A total of 69 specifically identified DEGs were down-regulated in the fertile plants of P1. Of them, 53 DEGs have known function, and most of them are related to cell or cell wall development. For instance, three and two DEGs were encoding callose synthases and laccases, respectively. It is interesting to found that a DEG encoding suppressor of auxin resistance was down-regulated in the fertile plants of P1, and this implied that the signal pathway of auxin might play a role in *Rf3* mediated fertility restoration of CMS-S.

In P2, function of two specific DEGs is unknown, six of the ten rest DEGs are up-regulated in the fertile plants. These DEGs encode two P450 proteins, an expansin, a SLAC, a ULP1B, and a leguminous lectin. Four DEGs encoding a protein phosphatase, an oligopetide transporter, a heat stress transcription factor, and a benzoxazinone synthesis, respectively, were down-regulated in the fertile plants of P2.

### Function of the DEGs co-expressed in P1 and P2

All of the 265 DEGs simultaneously expressed in the two populations were up-regulated in the fertile plants, and 54 of them have unknown function. A number of the annotated DEGs are involved in cell wall expansion. These include 20 DEGs encoding pectin methylesterases and pectin methylesterase inhibitors (PME/PMEIs), which plays a role in plant cell wall modification and subsequent breakdown (Carpita & Gibeaut, 1993); 19 DEGs encoding expansins, which are cell wall proteins inducing cell wall loosening and participate in all plant growth and development processes (Campbell & Braam, 1999); and ten DEGs encoding pectatelyases and 13 DEGs for polygalacturonases, which play a role in the breakdown of pectin (Jiang, Lu & Imsabai, 2008). In addition, DEGs encoding three lipase transfers (GDSL), two methyltransferases, five allergens, two cellulose synthases, four fasciclin-like arabinogalactan proteins, and a glyoxal oxidase are also up-regulated in the fertile plants of the two populations. These genes have been also found to be involved in the development of cell wall in plants (Li et al., 2010; Hu et al., 2014; Zhang et al., 2016; An et al., 2019). RAPID ALKALINIZATION FACTOR (RALF) peptides could be as sensors of cell wall integrity (Vogler et al., 2019). Four DEGs encoding RALFs were found to be up-regulated in the fertile plants of the two populations.

Three DEGs encoding glycosyl hydrolases and a DEG for UDP-glycosyltransferase were also co-expressed in the two populations, and these genes are involved in the process of carbohydrate metabolism. Plant cation/H^+^ exchangers (CHXs) are transporters that modulate K^+^ and pH homeostasis and are important for saccharide transport. A total of 11 DEGs encoding cation/H^+^ exchangers, H^+^-ATPase, cation-chloride co-transporter, a major intrinsic protein (MIP), a sucrose transporter, and oligopeptide transporter were co-expressed in the two populations. Seven DEGs encoding cysteine-rich proteins were up-regulated in the fertile plants of the two populations. One of them encodes a CAP (cysteine-rich secretory proteins antigen 5 and pathogenesis-related protein 1). It is interesting to find that five of the identified cysteine-rich proteins have paired CXXXC motifs which cluster on the long arm of chromosome 2 harboring *Rf3*. Peroxidase (POD) is one of the antioxidant enzymes which could reduce the level of ROS in plant cells. In this study, three co-expressed DEGs encoding POD were highly expressed in the fertile plants. DEGs encoding four pollen calcium-binding proteins, a transmembrane BAX inhibitor protein, three aspartic proteinases, a cysteine proteinase, and two proteinase inhibitors were co-expressed in the two populations.

In addition, genes related transcription factor and signal transduction were also found to be up-regulated in the fertile plants of the two populations. DEGs for two LRR, a bHLH (basic helix-loop-helix), four zinc fingers, three Leucine-rich repeat (LRR) family proteins, a WD40 repeat protein, a GASR3 (Gibberellin-regulated GASA/GAST/Snakin family proteins), 20 protein kinases were co-expressed in the two populations.

Another 61 DEGs related to cell growth and energy metabolism were also co-expressed in the two populations, including genes for actin depolymerizing factors, plasma membrane ATPase, and electron transport. Such as, three DEGs, which encode a cytochrome c (*GRMZM5G856180*), a NADH-ubiquinone reductase (*GRMZM6G602171*) and a NAD(P)-dependent oxidoreductase (*GRMZM2G453311*), are involved in electron transport chain and energy metabolism. NADH dehydrogenase and cytochrome c are the components of the mitochondrial respiratory chain, and any disturbance of this electron transport chain would influence the energy production.

## Discussion

### Majority of the DEGs identified in the two populations are related to anther and pollen development

Cell wall expansion is important during the maturation of anthers and pollens, and mutation of genes related to cell wall expansion would result in male sterility (Zhang et al., 2010; Campbell & Braam, 1999). In this study, of the 265 DEGs simultaneously up-regulated in the fertile plants of the two populations, a total of 62 DEGs (20 for PEM/PMEIs, 19 for expansions, ten for pectatelyases, and 13 for polygalacturonases) are related to cell wall expansion. Polygalacturaonase was found up-regulated in the fertile line in the stages of pollen grain maturation and pollen tube growth (Jiang, Lu & Imsabai, 2008). PME/PMEI genes were also found highly expressed in male-fertile lines in comparison with their sterile counterparts in *Brassica napus* (Zhu et al., 2010), cotton (Suzuki et al., 2013), and maize (Zhang et al., 2019). In addition, silencing of an *Arabidopsis* PMEI gene, *At1g10770*, could result in male sterility (Zhang et al., 2010). These imply that the genes participating in plant cell wall modification and loosening are important for anther and pollen development.

Callose wall and primexine formation is important during microsporogenesis, and mutation of these genes would also affect pollen fertility. Exine, the outermost layer of a pollen grain, has important roles in protecting microspore cytoplasm and determining species-specific interactions between pollen and stigma. The Exine Formation Defect (EFD) gene encodes a methyltransferase, and it was involved in both callose wall and primexine formation during microsporogenesis (Hu et al., 2014). *ZmMs30* encodes a GDSL lipase, which participates in anther cuticle and pollen exine development, and was essential for male fertility (An et al., 2019). Glycosyl hydrolase is responsible for catalyzing the hydrolysis of *β*-1,3-glucan, and it may be related to the degradation of callose. Glycosyl hydrolase family 17 protein (A6) was found to be irreplaceable in anther and pollen development (Xu et al., 2010). In this study, DEGs for methyltransferases, glycosyl hydrolases, and GDSL lipases were also detected significantly, highly expressed in the fertile plants of the two populations. In addition, of the three co-expressed DEGs encoding glycosyl hydrolases, *GRMZM2G005798* is a homolog of the A6 gene. Allergenic proteins are located in the coat of pollens are a major cause of allergic reaction, and some allergenic proteins were down-regulated in triploid sterile poplar pollens (Zhang et al., 2016). Fasciclin-like arabinogalactan proteins (FLA) are predominantly located in the intercellular spaces. In *Arabidopsis*, abnormal pollens were observed in FLA3-RNA interference transgenic plants, and intine layer of the defect pollens was affected (Li et al., 2010). RALF peptides can mediate the communication between cell wall components and plasma membrane-localized receptor-like kinases (RLKs) during pollen tube growth (Vogler et al., 2019). In this study, DEGs for five allergic proteins, four FLAs, and four RLKs were identified in the two populations. Furthermore, the DEGs encoding two cellulose synthases, four arabidnogalactan, and a glyoxal oxidase were also up-regulated in the fertile plants. They might be also involved in the cell wall development in maize.

Co-expressed DEGs related to carbohydrate metabolism and energy metabolism, such as that encode two sugar transporters, two UDP-glycosyltransferases, a cytochrome c, and a NADH-ubiquinone reductase, were identified in this study. In addition, many co-expressed DEGs encoding transporters, LEA, HAK and cation/H^+^ exchangers (CHXs) were up-regulated in the fertile plants. These genes are important for anther and pollen development, defect on this gene would affect male fertility (Stadler et al., 1999; Sivitz, Reinders & Ward, 2008). In rice, OsHAK1 knockout mutants had effect on root growth, pollen viability, and fertility, accompanied by a down-regulation of genes encoding sucrose transporters (SUT genes) and monosaccharide transporters (MST genes) (Chen et al., 2018). AtCHX17, AtCHX18, and AtCHX19 are membrane transporters that modulate K^+^ and pH homeostasis, loss of function would affect pollen wall formation, male fertility, and embryo development (Padmanaban et al., 2017). Sucrose transporter (AtSUC1) was highly expressed in pollen, and it was important for anther dehiscence and pollen tube growth in *Arabidopsis* (Stadler et al., 1999; Sivitz, Reinders & Ward, 2008).

Programmed cell death (PCD) is involving in the development of tapetum and pollen. It plays important roles in normal degradation of tapetum cells and the release of microspores from tetrads. In the maize CMS-S line, PCD was found to take place earlier than its restorer line (Mu et al., 2006). Expression of AtBAG6, an *Arabidopsis* CaM-binding protein that contains a BCL-2-associated athanogene (BAG) domain and an IQ motif (IQXXXRGXXXR), was specifically induced by agents generating oxygen radicals, and overexpression of AtBAG6 induced cell death phenotypes (Kang et al., 2006). BAX is BCL2-associated proteins, and Bax Inhibitor-1 (BI-1) is a Ca^2+^ channel involved in regulating intracellular Ca^2+^ homeostasis. Overexpression of *Arabidopsis* Bax inhibitor-1 (AtBI-1) was able to suppress Bax-, hydrogen peroxide-, and salicylic acid-mediated cell deaths (Kawai-Yamada, Ohori & Uchimiya, 2004). Proteinases/proteinase inhibitors are also related to PCD. For instance, bvORF20, strong homology with the OMA1-like metallopeptidase, would restore fertility post-translationally in sugar beet (*Beta vulgaris* L.) (Kitazaki et al., 2015); PROMOTION OF CELL SURVIVAL1 (PCS1) encodes an aspartic protease, and the progression of PCD degeneration in the anther appears to be halted when PCS1 is ectopically expressed under regulation of the cauliflower mosaic virus (CaMV) 35S promoter in Arabidopsis (Ge et al., 2005). In rice, two aspartic proteases, OsAP25 and OsAP37, regulated by ETERNAL TAPETUM 1, also were found to play roles in male fertility by regulating tapetal cell death during male reproductive development (Niu et al., 2013) or pollen germination and pollen tube growth (Huang et al., 2013). Transcription factors have been reported played roles on male fertility in plants. For instance, two bHLH transcription factors, Ms23 and Ms32, act with the HD-ZIP transcription factor OCL4 to regulate the differentiation and development of somatic anther wall layers (Vernoud et al., 2009; Moon et al., 2013; Nan et al., 2017). ETERNAL TAPETUM 1, a bHLH transcription factor, was found to be related to tapetal cell death and aborted pollen formation in rice (Niu et al., 2013). WD40 repeat proteins facilitate protein-protein interactions and function as scaffold proteins for the formation of multiprotein complexes. A WD40 and a RNA binding protein, GRP162 were required in the fertility restoration complex in HL rice by interaction with RF5 (Qin et al., 2016). In this study, DEGs for seven proteases (including three aspartic proteases), five calcium-binding proteins (including two proteins with BAX inhibitor motif or IQ motif), three PODs, a WD40 repeat protein, and a bHLH were identified in the two populations. These DEGs might have to do with male fertility restoration for CMS-S via PCD.

Thus, the 265 co-expressed DEGs are mainly related to cell wall development, PCD process and energy metabolism. Mutations of these genes were found to lead to male-sterile in different plant species (Hu et al., 2014; An et al., 2019; Xu et al., 2010; Stadler et al., 1999; Niu et al., 2013; Huang et al., 2013). In addition, 227 (85.7%) of them were specifically or mainly expressed in pollen or anther (http://qteller.maizegdb.org/). This indicates that these DEGs might be involving the development of anther or pollen in maize.

### JA signal might be involved in the *Rf3* mediated fertility restoration to CMS-S

A PPR gene in the mapping region might be the candidate of *Rf3* (Feng et al., 2015), however, the mechanism of fertility restoration of CMS-S is unknown. In this study, 22 DEGs specifically and up-regulated in the fertile plants of the population with *Rf3* were identified. These DEGs would give clues for unveiling the mechanism of *Rf3* mediated fertility restoration of CMS-S.

Jasmonic acid (JA) is an important plant hormone regulating plant growth and development. Studies on the mutants of genes related to JA synthases or sensitive to JA revealed that JA is related to anther dehiscence and pollen fertility (Stintzi & Browse, 2000). Jasmonat-zim-domain protein (JAZ) could repress the transcription of JA-responsive genes, and the degradation of JAZ would allow JA signal transduction (Devoto et al., 2002; Katsir et al., 2008). In this study, a DEG encoding JAZ1 was specifically up-regulated in the fertile plants of P1. JA-repressors, JAZ1 and JAZ8, were found down-regulated in the transgenic *Arabidopsis* plants expressing GhWRKY22, and anther/pollen development in the over-expression plant was affected (Wang et al., 2019). JAZ1 could bind to ENHANCER OF GLABRA3 (EGL3), a bHLH component of the WD-repeat/bHLH/MYB complex, and the DELLA protein RGA competed with JAZ1 to bind to EGL3 (Tian et al., 2016). This suggests cross-talk between the JA and GA pathways is involved in the pollen development, and this is consistent with the fact that a DEG encoding GASR3 was up-regulated in the fertile plants. In addition, a DEG encoding WD40-repeat protein was also up-regulated in the fertile plants, implying that WD40/bHLH/MYB complex might be also participated in the regulation of fertility restoration of CMS-S.

Jasmonyl-isoleucine (JA-Ile) is a major active compound in JA signaling. CYP94C1 is involved in the oxidation of JA-Ile, resulting in inactivating of JA-Ile (Bruckhoff et al., 2016). It is interesting to find that of the two DEGs for P450 proteins specifically up-regulated in the fertile plants of P1, *GRMZM2G159179* was predicted to be a homolog of *CYP94C1*. Furthermore, previous studies have shown that thionins could be regulated by JA, and are important for plant fertility (Ishiguro et al., 2001; Nakata & Ohme-Takagi, 2013; Yuan & Zhang, 2015). CaThi, a thionin-like protein isolated from the fruit of *Capsicum annuum* could trigger apoptosis in *C. tropicalis* cells (Taveira et al., 2018). MdD1, a gamma-thionin protein from apple, was shown to bind to the RNase activity sites of S-RNase leading to inhibition of pollen tube growth (Gu et al., 2019). In this study, two DEGs encoding thionins were identified, and one of them (*GRMZM2G339781*) specifically up-regulated in the fertile plants of P1.

Protein kinases play roles in signal transduction. Mutants of two receptor-like cytoplasmic kinases (RLCK) in maize, ZmSTK1 or ZmSTK2, exhibited severe pollen transmission deficiency and fertility (Fan et al., 2018). In *Arabidopsis*, suppression of ASK *β* (AtSK32), a Clade III Arabidopsis GSK3, leaded to pollen defect during late pollen development (Dong et al., 2015). Fertility of two mutants of calcium/calmodulin-dependent kinases (Ca^2+^/CaMKs), *camk-1* and *camk-2*, was affected in *Neurospora. crassa* (Kumar & Tamuli, 2014). In P1, five DEGs encoding protein kinases were up-regulated in the fertile plants, and one of them is calcium/calmodulin-dependent protein kinase (CaMK, AC203294.3_FG001).

**Figure 5:**
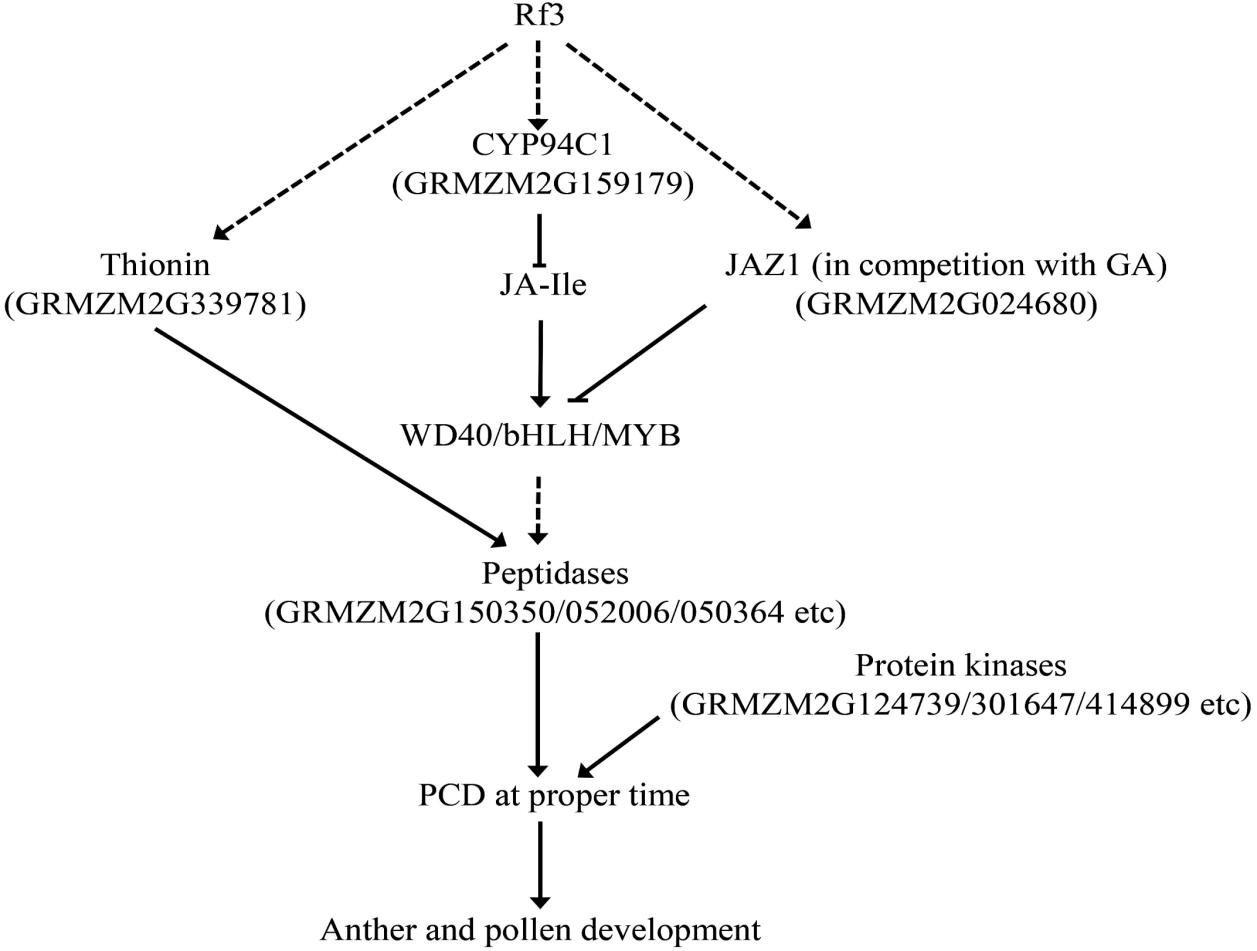
Some DEGs might be involved in the *Rf3* mediated fertility restoration of CMS-S in maize. The dotted lines mean the pathway is not clear.

Taken together, *Rf3*, interacting with *orf355-orf77*, might trigger PCD at the proper time via genes related to JA signal. Up-regulated JAZ1 and inactivation of JA-Ile (via up-regulated CYP94C1) could facilitate JAZ1 to bind the WD40/bHLH/MYB complex in competition with GA. This would interrupt the signal transduction of JA and GA, and subsequently induce PCD via increasing the expression of some protein kinases and peptidases (Fig. 5). In addition, expression of two important DEGs might be involved in this process was verified by qPCR (Fig. S3).

### The SUMO system might be involved in *Rf10* mediated fertility restoration to CMS-S

There are five DEGs only up-regulated in the fertile plants of *Rf10*, and none of them encoding PPR protein. This is much less than that for *Rf3*, suggesting the mechanism of fertility restoration of *Rf10* might be simpler. There is no any PPR gene located in the region harboring *Rf10*, thus fertility restoration of *Rf10* is not mediated by PPR protein. In sugar beet (*Beta vulgaris* L.), restoration of fertility of the Owen CMS is also not related to PPR protein (Matsuhira et al., 2012).

In this study, a DEG encoding an UB-like protein 1B (ULP1B) was specifically expressed in the fertile plants with *Rf10* (Fig. 3). ULP1B is a member of small ubiquitin-related modifier (SUMO) and it is one of the proteases responding for removing SUMO from the conjugated proteins. Two SUMO proteases, SUMO PROTEASE RELATED TO FERTILITY1 (SPF1) and SPF2, were found to be associated with male and female gamete and embryo development in *Arabidopsis* (Liu et al., 2017). In rice, defects in anther dehiscence, pollen fertility, and seed set percentage were observed in the mutant of a SUMO E3 ligase, which is another component of the SUMO system (Pei et al., 2019). Thus, it can be deduces that *Rf10* might restore the fertility of CMS-S via the SUMO system. This also indicates that multiple mechanisms might be involved in the fertility restoration of CMS-S (Feng et al., 2015).

## Conclusions

In this report, BSR-Seq was conducted using two backcrossing populations. Genetic analysis and BSR-Seq mapping revealed that two main fertility restoration genes of maize CMS-S, *Rf3* and *Rf10*, exclusively existed in the two populations. A total of 353 and 176 DEGs were identified in the populations with *Rf3* and *Rf10*, respectively. Of them, 265 DEGs were co-expressed and up-regulated in the fertile plants of the two populations, and 35 and 7 DEGs were specifically up-regulated in the fertile plants of the population with *Rf3* and *Rf10*, respectively. Expression profile of some DEGs in the two populations was verified through qPCR. Function analysis of these DEGs indicated that majority of the DEGs might be involving in anther or pollen development, and JA signal pathway and small ubiquitin-related modifier system could be involving in *Rf3* and *Rf10* mediated fertility restoration of CMS-S. Our study would provide useful information for the study on male-sterile and the mechanism of fertility restoration of maize CMS-S.

##  Supplemental Information

10.7717/peerj.10015/supp-1 Supplemental Information 1 The raw data-rf3 Rf3 Rf10 qPCR cq number

10.7717/peerj.10015/supp-2 Figure S1 Pollen fertility in the sterile (with un-exserted anthers) and fertile plants (with exserted anthers) of the two populations

10.7717/peerj.10015/supp-3 Figure S2 Comparison of the expression of the 18 DEGs between the analyses of Q-PCR and BSR-Seq P-S: sterile plants/pool in P1 and P2; P1-F: fertile plants/pool in P1; P2-F: fertile plants/pool in P2. A-B: The twelve DEGs up-regulated in the fertile plants of the two populations. C: The three DEGs specifically up-regulated in the fertile plants of P1 or P2. D: The three DEGs specifically down-regulated in the fertile plants of P1 or P2. The lines and dotted lines with different colors represent the expression of different DEGs from the analyses of Q-PCR and BSR-Seq, respectively.

10.7717/peerj.10015/supp-4 Figure S3 Quantitative RT-PCR confirmation ofthe two importantDEGs might be involving in *Rf3* mediated fertility restoration of CMS-S Genotypes of *rf* and *Rf3* are S-CMS^*rf3rf3rf10rf10*^ and S-CMS^*Rf3rf3rf10rf10*^,respectively.** represents the difference of relative expression between*Rf3* and *rf* are significant at the level of *P* < 0.01 (*n* = 3).

10.7717/peerj.10015/supp-5 Figure S4 Specific amplification of primers detected by agarose electrophoresis Odd number lane mean PCR amplification Include template; even numbers lane mean no template PCR. First row: Lane1-2:*GRMZM2G024887*; Lane3-4:*GRMZM2G072448*; Lane5-6;*GRMZM2G300969*; Lane7-8;*GRMZM2G347047*; Lane9-10:*GRMZM2G390678*; Lane11-12;*GRMZM2G401874*; Lane13-14;*GRMZM2G419452*; Lane15-16;*GRMZM2G454608*; Lane17-18:*GRMZM2G045030*; Lane19-20;*GRMZM2G109973*; Lane21-22;*GRMZM2G339781*; Lane13-14;*GRMZM2G435294*. Seconed row: Lane1-2:*AC209946.4-FG001*; Lane3-4:*GRMZM2G020761*; Lane5-6;*GRMZM2G024680*; Lane7- 8;*GRMZM2G127418*; Lane9-10:*GRMZM2G322493*; Lane11-12;*GRMZM2G457789*; Lane13-14; *LOC100282267*

10.7717/peerj.10015/supp-6 Table S1 DEGs_in_P1_and_P2

10.7717/peerj.10015/supp-7 Table S2 qPCR gene primer sequence

## Additional Information and Declarations

### Data Availability

The following information was supplied regarding data availability: The raw data are available in Supplemental File.
