# Supplementary material for: Identification of fertility-related genes for maize CMS-S via Bulked Segregant RNA-Seq
Source: PeerJ. 2020 Sep 30;8:e10015. doi: 10.7717/peerj.10015 (PMC7532766; doi:10.7717/peerj.10015)
Supplement: Figure S1 [file peerj-08-10015-s002.pdf]

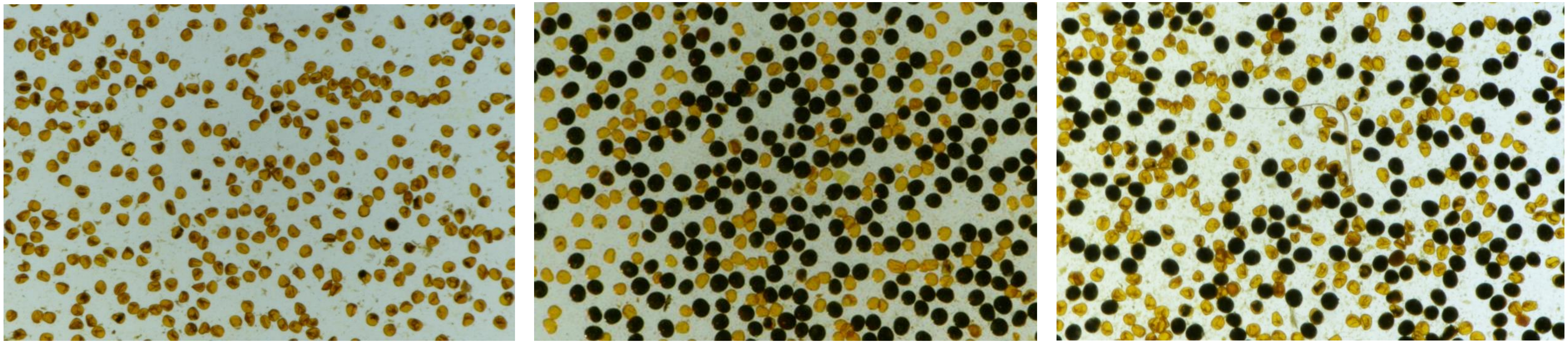

S-CMS^{rf3rf3rf10rf10} S-CMS^{Rf3rf3rf10rf10} S-CMS^{rf3rf3Rf10rf10}

Figure S1 pollen fertility in the sterile (with un-exserted anthers) and fertile plants (with exserted anthers) of the two populations
