# Supplementary figures and images for: Identification of fertility-related genes for maize CMS-S via Bulked Segregant RNA-Seq
Source: PeerJ. 2020 Sep 30;8:e10015. doi: 10.7717/peerj.10015 (PMC7532766; doi:10.7717/peerj.10015)

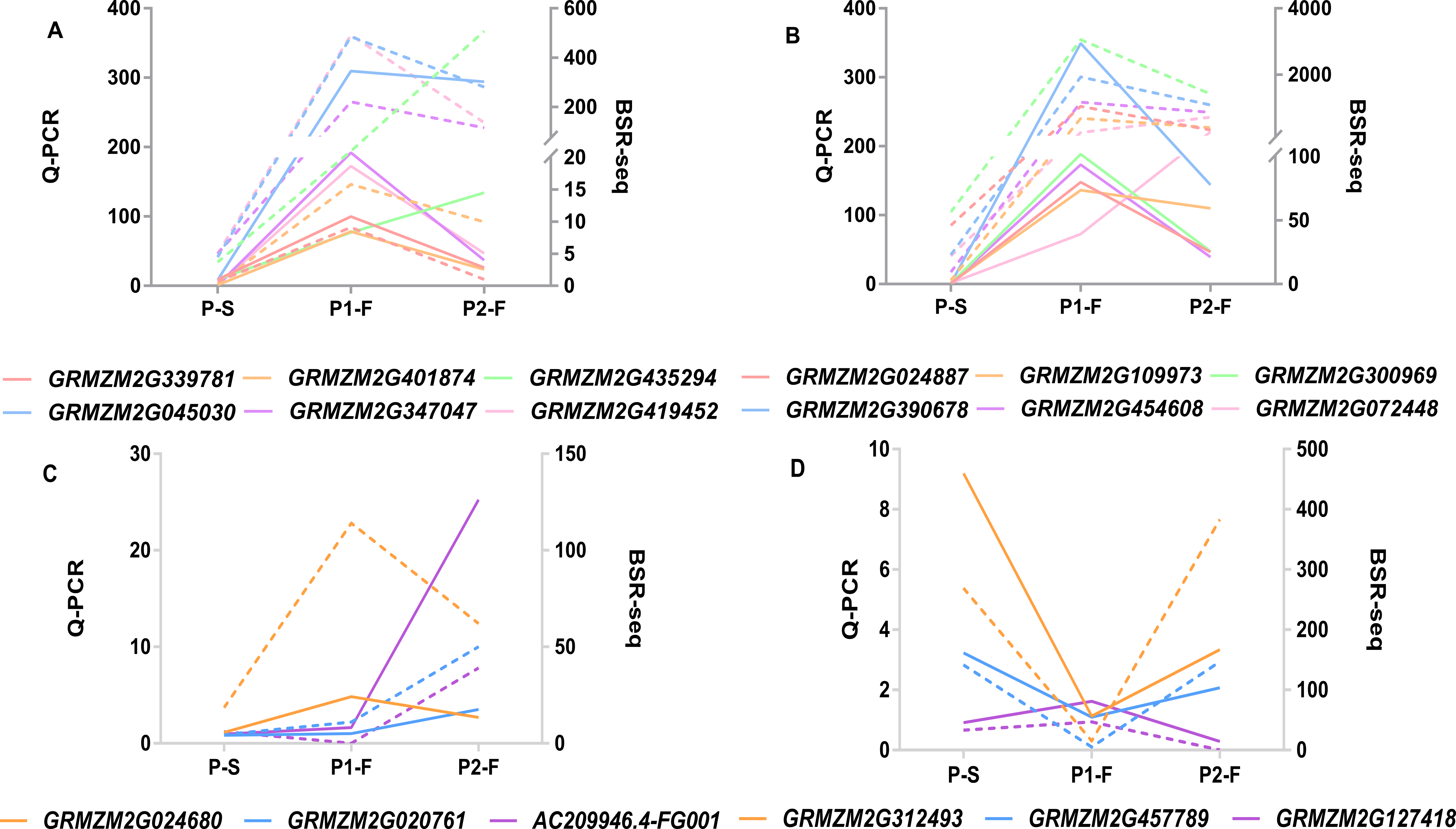

Supplement: Figure S2 — P-S: sterile plants/pool in P1 and P2; P1-F: fertile plants/pool in P1; P2-F: fertile plants/pool in P2. A-B: The twelve DEGs up-regulated in the fertile plants of the two populations. C: The three DEGs specifically up-regulated in the fertile plants of P1 or P2. D: The three DEGs specifically down-regulated in the fertile plants of P1 or P2. The lines and dotted lines with different colors represent the expression of different DEGs from the analyses of Q-PCR and BSR-Seq, respectively. [file peerj-08-10015-s003.png]

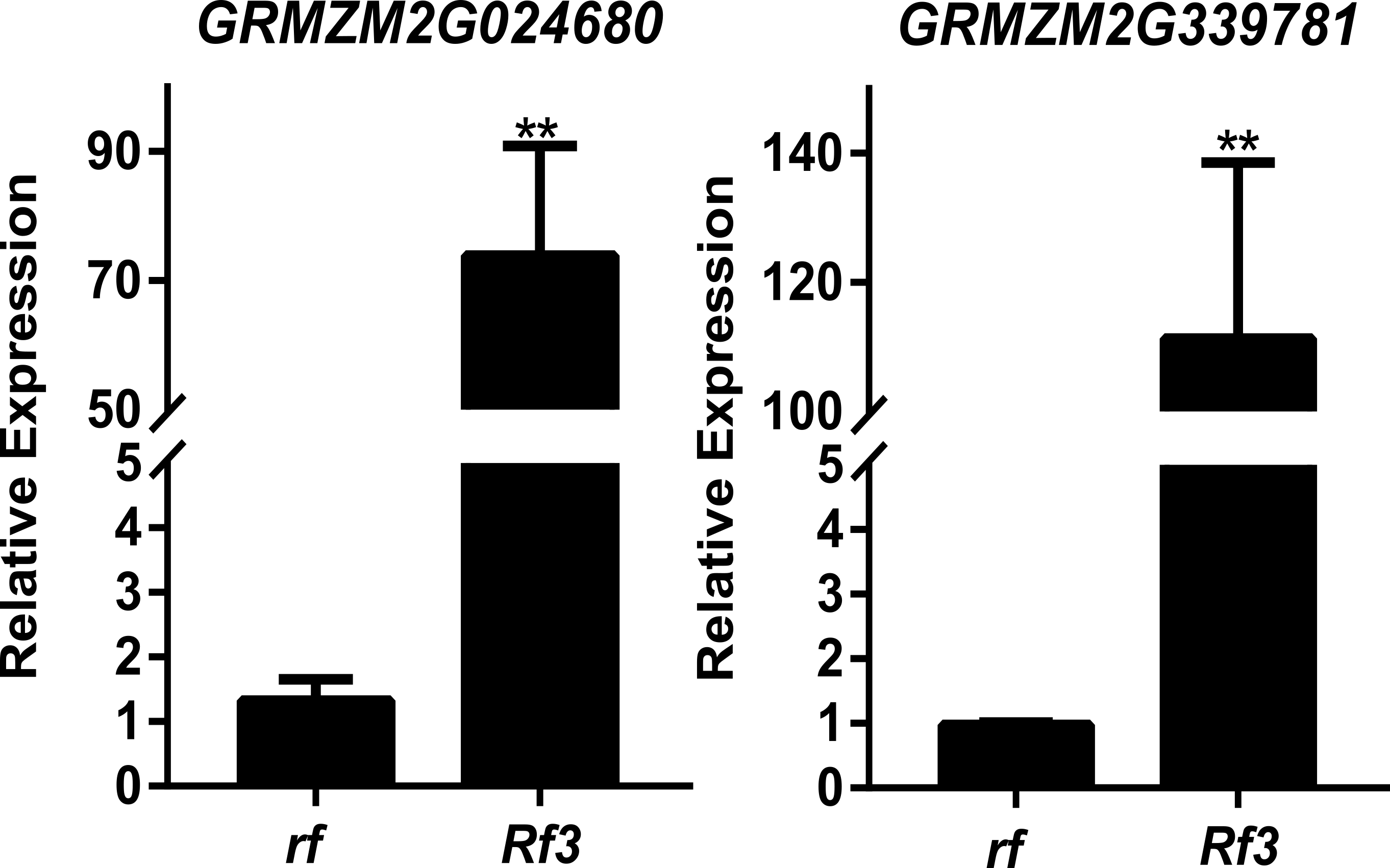

Supplement: Figure S3 — Genotypes of rf and Rf3 are S-CMSrf3rf3rf10rf10 and S-CMSRf3rf3rf10rf10,respectively.** represents the difference of relative expression betweenRf3 and rf are significant at the level of P < 0.01 (n = 3). [file peerj-08-10015-s004.png]
