# Supplementary material for: Identification of fertility-related genes for maize CMS-S via Bulked Segregant RNA-Seq
Source: PeerJ. 2020 Sep 30;8:e10015. doi: 10.7717/peerj.10015 (PMC7532766; doi:10.7717/peerj.10015)
Supplement: Figure S4 — Odd number lane mean PCR amplification Include template; even numbers lane mean no template PCR. First row: Lane1-2:GRMZM2G024887; Lane3-4:GRMZM2G072448; Lane5-6;GRMZM2G300969; Lane7-8;GRMZM2G347047; Lane9-10:GRMZM2G390678; Lane11-12;GRMZM2G401874; Lane13-14;GRMZM2G419452; Lane15-16;GRMZM2G454608; Lane17-18:GRMZM2G045030; Lane19-20;GRMZM2G109973; Lane21-22;GRMZM2G339781; Lane13-14;GRMZM2G435294. Seconed row: Lane1-2:AC209946.4-FG001; Lane3-4:GRMZM2G020761; Lane5-6;GRMZM2G024680; Lane7- 8;GRMZM2G127418; Lane9-10:GRMZM2G322493; Lane11-12;GRMZM2G457789; Lane13-14; LOC100282267 [file peerj-08-10015-s005.pdf]

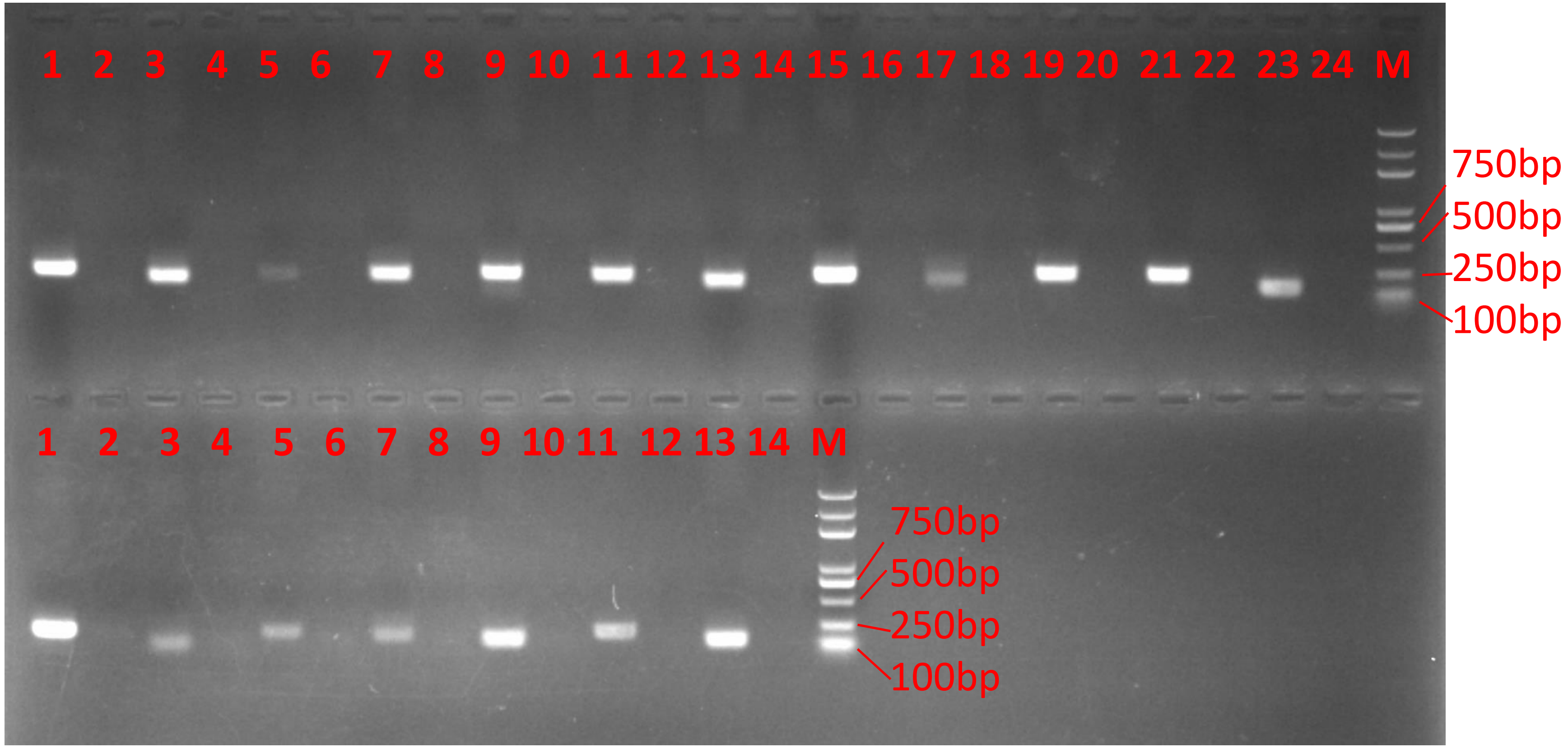

Figure S4: Odd number lane mean PCR amplification Include template; even numbers lane mean no template PCR.
First row: Lane1-2:*GRMZM2G024887*; Lane3-4:*GRMZM2G072448*; Lane5-6;*GRMZM2G300969*; Lane7-8;*GRMZM2G347047*; Lane9-10:*GRMZM2G390678*; Lane11-12;*GRMZM2G401874*; Lane13-14;*GRMZM2G419452*; Lane15-16;*GRMZM2G454608;* Lane17-18:*GRMZM2G045030*; Lane19-20;*GRMZM2G109973*; Lane21-22;*GRMZM2G339781*; Lane13-14;*GRMZM2G435294*.
Seconed row: Lane1-2:*AC209946.4-FG001*; Lane3-4:*GRMZM2G020761*; Lane5-6;*GRMZM2G024680*; Lane7-8;*GRMZM2G127418*; Lane9-10:*GRMZM2G322493*; Lane11-12;*GRMZM2G457789*; Lane13-14; *LOC100282267*
